# Effect of Lactic Acid Etching on Bonding Effectiveness of Orthodontic Bracket after Water Storage

**DOI:** 10.1155/2014/719608

**Published:** 2014-03-17

**Authors:** Fahad F. Alsulaimani

**Affiliations:** Division of Orthodontics, Department of Preventive Dental Science, Faculty of Dentistry, King Abdulaziz University, Jeddah, Saudi Arabia

## Abstract

*Objective*. To determine the effect of lactic acid at various concentrations on the shear bond strength of orthodontic brackets bonded with the resin adhesive system before and after water storage. *Materials and Methods*. Hundred extracted human premolars were divided into 5 treatment groups and etched for 30 seconds with one of the following agents: lactic acid solution with (A) 10%, (B) 20%, (C) 30%, and (D) 50%; group E, 37% phosphoric acid (control). Metal brackets were bonded using a Transbond XT. Bonding effectiveness was assessed by shear bond strength after 24 hours and 6 months of water storage at 37°C. The data were analyzed with 2-way analysis of variance and Tukey's Honestly Significant Difference (HSD) test (α = .001). *Results*. Lactic acid concentration and water storage resulted in significant differences for brackets bond strength (*P* < .001). 20% lactic acid had significantly higher mean bond strength values (SD) for all conditions: 24 hours [12.2 (.7) MPa] and 6 months [10.1 (.6) MPa] of water storage. 37% phosphoric acid had intermediate bond strength values for all conditions: 24 hours [8.2 (.6) MPa] and 6 months [6.2 (.6) MPa] of water storage. Also, there were differences in bond strength between storage time, with a reduction in values from 24 hours and 6 months for all experimental groups (*P* < .001). *Conclusion*. Lactic acid could be used in place of phosphoric acid as an enamel etchant for bonding of orthodontic brackets.

## 1. Introduction

The efficacy of various agents as an enamel etchants during bonding of orthodontic brackets has been studied [1–11]. Nevertheless, phosphoric acid has remained the principal enamel etchant since it was first introduced by Buonocore [12] in 1955 and used by Newman [13] in 1965. Phosphoric acid concentration between 30% and 40% results in the most retentive etching pattern [14, 15].

Acid etching of enamel is recommended with many dentinal bonding systems to improve the delivery of orthodontic treatment [1]. Claims of comparable bond strengths to enamel and dentin with conventional methods of bonding have meant that these adhesives are also suitable for orthodontic bonding [15–19]. The development of an adhesive bond requires establishing intimate contact between the liquid adhesive and the solid adherent, minimizing the stress concentration at the interface and reducing the influence of environmental factors on the interface integrity [20–22]. Therefore, the materials which are used today to adhere to the hard tooth structure must resist the surrounding influences in the oral cavity, including temperature changes [23]. Moreover, the bond strength of adhesive and attachments should be sufficient to withstand all forces and stresses exerted by mastication and archwires. Although there is no formally accepted minimum clinical bond strength, 5 to 10 MPa have been mentioned as being adequate for clinical situations [2–5].

Long-term stability of bonding to tooth structure remains questionable. A factor known to promote bond degradation is long-term water exposure [6, 7, 24–26]. Bond deterioration by water storage might be caused by degradation of interface components, such as denaturation of collagen and/or elution of degraded or insufficiently cured resin [23, 27]. Therefore, maintaining a sound unblemished enamel surface is a primary clinical concern when debonding the brackets after orthodontic treatment. Enamel fracture and cracks have been reported at the time of bracket debonding [28]. It is possible that the depth of the etched enamel surface created by phosphoric acid may be a contributing factor to the incidence of enamel fracture [29]. Therefore, to minimize the extent of enamel surface damage, alternative conditioners, such as maleic acid [30, 31], nitric acid [32], polyacrylic acid [33], and ethylenediaminetetraacetic acid [34], have been used to obtain clinically useful bond strengths by decreasing the depth of enamel dissolution.

Mount [35] suggested that etchants should be (1) isotonic to avoid osmotic pressure changes, (2) neutral pH or at least between pH 5.5 and pH 8.0, (3) nontoxic to dentin, pulp, and gingival tissue, (4) compatible with the chemistry of the cementation agents, (5) water soluble and easily removed, (6) unable to deplete the enamel or dentin chemically, and (7) able to enhance the surface chemically in preparation for bonding. Although none of the currently available dentin etchants meet all these requirements, it would seem desirable to use a conditioning agent that had the least possibility of causing adverse reactions. Because lactic acid occurs naturally in the muscles under anaerobic conditions during muscular exercise, it is thought to be more biologically acceptable than other etchants [36, 37]. However, its action as an alternative to phosphoric acid as enamel and dentin etchant has not been reported. The purpose of the present study was to determine the effect of lactic acid at various concentrations on the shear bond strength of orthodontic brackets bonded with Transbond XT resin adhesive at 24 hours and 6 months of water storage. The null hypothesis was that lactic acid etchant at different concentrations would have no influence on the shear bond strength of orthodontic brackets before and after water storage.

## 2. Materials and Methods

The study protocol was approved by the Institutional Research Board. The pH of the tested etching solutions was measured with a Corning pH meter (model 220, Corning Science Products, Corning, NY) equipped with Corning combination electrode. The pH reference solutions at pH 7 and 4 were used to standardize the electrode. The average of three repeated measurements was determined.

Hundred intact recently extracted human upper premolars were debrided to remove remnants of periodontal ligaments and examined stereoscopically at ×10 to verify the absence of cracks. The teeth were stored in distilled water with 0.1% thymol disinfectant (Mallinckrodt Baker Inc., Phillipsburg, NJ) at room temperature and used within 1 week after extraction. Crowns of the selected teeth were sectioned perpendicular to the long axis, 5 ± 1 mm apical to the cementoenamel junction with a 0.15 diamond wafering blade (Buehler, Lake Bluff, IL) in an Isomet 1000 slow speed saw (Isomet, Buehler) under copious water cooling. Each crown was aligned horizontally in an individual polymeric tube (Buehler) and embedded in epoxy resin (Epoxide Resin, Leco Corp., St. Joseph, MI) so that the buccal surfaces projected well above the embedding material. A surveyor (J.M. Ney Co., Bloomfield, CT) was used to align the bonding surface perpendicular to the base of the mold. After complete polymerization, the specimens were polished to remove epoxy resin overflow. The buccal surfaces were cleaned with slurry of nonfluorinated pumice and water for 10 seconds, washed, and gently dried with an oil-free and moisture-free jet of air. Mounted teeth were stored in an atmosphere of 100% humidity.

The specimens were divided into 5 groups (*n* = 20 per group) and etched with 1 of the following agents: lactic acid solution with (A) 10%, (B) 20%, (C) 30%, and (D) 50% prepared at the Ohio State University, Department of Chemistry. The control group (E) was treated with 37% phosphoric acid (3 M Dental Products, Monrovia, CA). Etchant solutions were applied to enamel surfaces for 30 seconds with a cotton pledget and cotton pliers [14]. Immediately after etching, specimens were rinsed with water spray for 10 seconds in a direction that carried the acid away from the enamel surface. Transbond XT primer (3 M Unitek, Monrovia, CA) was applied on the etched enamel surfaces, and the metal maxillary premolar brackets (Mini-Taurus RMO, Denver, CO) were bonded onto the midbuccal surfaces using Transbond XT adhesive paste (3 M Unitek, Monrovia, CA) according to manufacturers' instructions. Transbond XT adhesive paste was applied to the base of the bracket which was then pressed firmly on to the tooth. Excess adhesive was removed from around the base of the bracket with a scaler and was light polymerized (UltraLume LED 5; Ultradent Products) for 10 seconds on each interproximal side at a 2 mm distance from each bracket face. Light intensity (730 mW/cm^2^) was monitored by a radiometer (Demetron/Kerr, Danbury, CT) before every use. The bonded teeth were left undisturbed for 30 minutes to ensure complete polymerization of the adhesive material. All bonding procedures were performed by the same operator.

**Figure 1:**
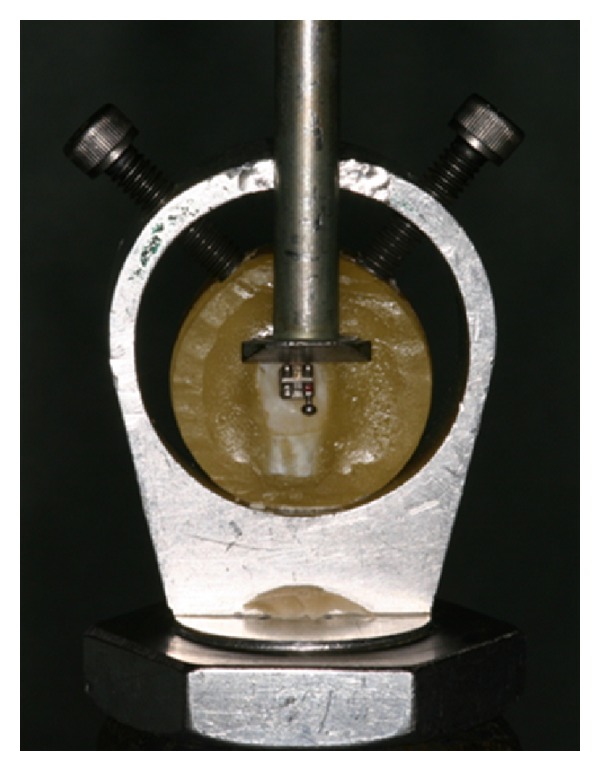
Special clamping device used in the study.

The failure loads for bonded brackets were determined on half of the specimens (*n* = 10 per group) after 24 hours, and the other half were stored in a hermetically sealed container with distilled water at 37°C to be tested after 6 months. Each specimen was locked in a special clamping device (Figure 1) mounted on the universal testing machine (Instron 4204; Instron Corp., Canton, MA). An occlusogingival load was applied to the area between the base and the wings of the bracket from a knife-edge fixture with a 1000 N load cell in the shear mode and a crosshead speed of 0.05 mm/min. The maximum loads were recorded and divided by the surface area of the bracket-enamel interface to obtain an estimate of the shear bond strength in megapascals (MPa).

An additional specimen from each group was treated and prepared for scanning electron microscopy (SEM) by mounting on aluminum stubs and sputter coating with very thin layer (approximately 1 nm) of gold-palladium alloy (Cressingtons sputter coater, Cressington Scientific Instruments Ltd., Watford, United Kingdom), and it was observed by a single investigator with a scanning electron microscope (Philips Electron Optics, BV. Achseweg Noords, The Netherlands) at the center of each specimen. Raster scans were performed at ×3000 magnification with an accelerating voltage of 15 KV. Mean values for each group were calculated, and differences between the groups were tested for statistical significance by use of 2-way analysis of variance and Tukey's Honestly Significant Difference (HSD) test (*α* = .001).

## 3. Results

The strength (pKa) of the tested etching acids and their pH measurements are listed in Table 1. The pH measurements for lactic acid were consistent and did not vary from the phosphoric acid.

One-way ANOVA for the results of shear bond strength revealed that there were significant differences between the group means (*P* < .001) (df 4, F 291.3, *P* < .001). Tukey's Honestly Significant Difference test disclosed a significant difference between each pair of surface treatments for each time of water storage.

The 2-way ANOVA results in Table 2 demonstrate a significant difference between the etchant (*P* < .001) and the storage times (*P* < .001). However, the interaction between etchant/storage time was not significantly different (*P* = .65). Mean values and standard deviations for each treated group are listed in Table 3. The highest mean shear bond strength (SD) was obtained from the group treated with 20% lactic acid solution on the 24 hours [12.2 (.7) MPa] and 6 months [10.1 (.6) MPa] of water storage. This is about 49% and 63% more than 37% phosphoric acid etchant on the 24 hours [8.2 (.6) MPa] and 6 months [6.2 (.6) MPa] of water storage. 50% lactic acid etchant had the lowest shear bond strength on the 24 hours [5.1 (.5) MPa] and 6 months [3.1 (.6) MPa] of water storage. This is about 61% and 100% less than 37% phosphoric acid etchant. There was a reduction in shear bond strength (21%–65%) after 6 months of water storage for all groups (*P* < .001).

**Figure 2:**
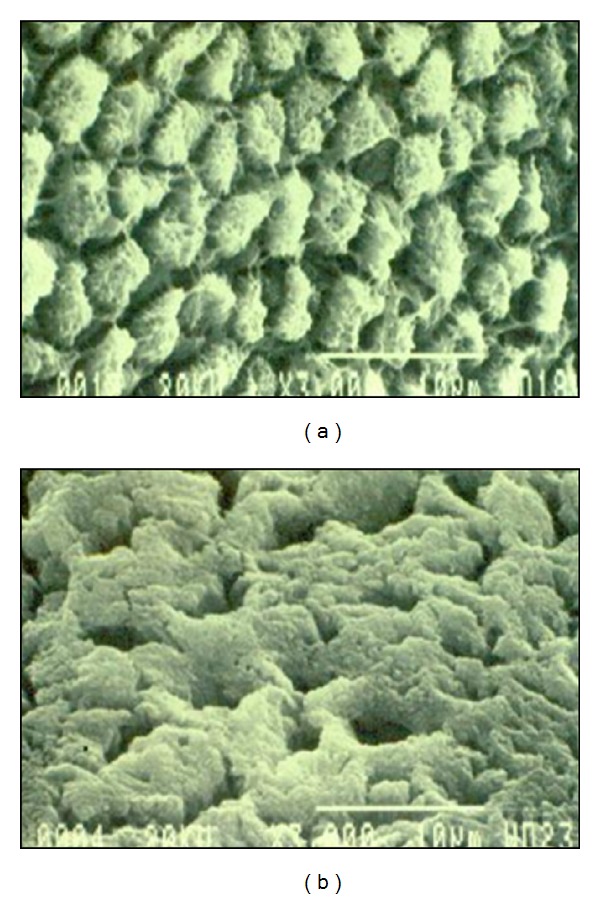
(a) SEM photomicrograph of enamel surface treated with 37% phosphoric acid for 30 seconds. (b) SEM photomicrograph of enamel surface treated with 20% lactic acid for 30 seconds.

Scanning electron microscopic (SEM) evaluation of the treated enamel surfaces of the specimens for 30 seconds revealed several types of morphological changes. The effect of 37% phosphoric acid illustrated in Figure 2(a), and the etching pattern obtained with 20% lactic acid is illustrated in Figure 2(b). SEM photomicrographs revealed it to be a well-defined apatitic-like crystal with intermittent interstices susceptible to resin penetration. Furthermore, etching increases the surface area and creates sites into which bonding agents can infiltrate and polymerize which results in adhesion.

## 4. Discussion

This study evaluated the effect of lactic acid surface treatment in different concentrations on shear bond strength of brackets bonded with the resin orthodontic adhesive system before and after water storage. The Transbond XT and metal brackets were employed in the current study due to their routine use at our dental school. Etch and bond technique using phosphoric acid is among those common bonding techniques used [2, 3, 15, 30, 32]. Premolars were used and the area of enamel surface used for bonding was similar to that used clinically when bonding orthodontic brackets. The bond strength of phosphoric acid etching has been shown to have clinically good values and the use of 5.25% sodium hypochlorite to eliminate the organic substances works together with it to increase the bond strength of the brackets-to-enamel surface [8].

Lactic acid etching is also among the techniques that have been suggested for treatment of enamel surface before being bonded [38]. The shear bond strength values of the current study were found to be near the clinically acceptable range recommended by Reynolds [5] who suggested a range of 5.9 to 7.8 MPa. The shear bond strength values of 6.1 MPa and 5.1 MPa presented with 30% and 50% lactic acid etchants for 24 hours of water storage and 4.1 MPa and 3.1 MPa for 6 months of water storage were statistically inferior to 37% phosphoric acid etchant (control) and were low in comparison with other researches [9–11]. However, the mean shear bond strength of 10% and 20% lactic acid was greater than the recommended values by Fritz et al. [17] to be adequate for routine clinical use.

Shinchi et al. [39] found that the adhesive strength of resin bonded to etched enamel depends mainly on the resin's ability to penetrate between the enamel crystallites and not necessarily to the depth of the enamel etched. In the current study, 20% lactic acid roughened the enamel surface by selective dissolution of prism cores or peripheries, creating micropores into which resin could flow [40]. However, to achieve reliable bond, the possibilities involve interaction with hydroxyapatite or collagen. Because hydroxyapatite is present in both enamel and dentin, it would seem most logical that the material adheres to hydroxyapatite.

Though the test method used in the present study attempted to simulate the clinical situation, there were some limitations. Bond strength was performed shortly after bonding (24 hours) and after water storage (6 months) without any simulations of oral condition. However, clinically, the dislodgement of brackets commonly occurs after several years of function, and long-term retention may be influenced by various factors such as temperature changes and dynamic mechanical loading. Another limitation of this study was that the site of failure was not observed. Furthermore, the current study was a laboratory investigation and in vivo research must be carried out to confirm laboratory results.

## 5. Conclusion

Within the limitations of this study, the following conclusions can be drawn.

1. Lactic acid etching could be used as an alternative to orthophosphoric acid etching when bonding orthodontic brackets with composite resin adhesive.
2. Significant time differences were observed between 20% lactic acid bond strength and the 37% phosphoric acid (control) regardless of the storage time.
3. Six months of water storage significantly reduced the shear bond strength regardless of the etchant used.

## Figures and Tables

**Table 1:** Strength of acids and their pHs*.

Etchant	pKa1	pKa2	pKa3	pH
37% phosphoric acid	2.12	7.21	12.67	0.16
10% lactic acid	3.86	—	—	1.55
20% lactic acid	3.86	—	—	1.40
30% lactic acid	3.86	—	—	1.20
50% lactic acid	3.86	—	—	1.07

*Power of hydrogen.

**Table 2:** Two-way repeated measure AVOVA.

Source of variation	df*	MS**	F score	*P* value
Groups	4	204.97	576.04	<.001
Time	1	110.84	311.49	<.001
Group ∗ time	4	.22	.62	.65
Error	90	.36		

*Degree of freedom, **mean square.

**Table 3:** Shear bond strength (MPa) for experimental groups according to the type of etchant [mean (SD*) *n* = 10].

Etchant	Storage time	
	24 hours of water storage^1^	6 months of water storage^2^
10% lactic acid^a^	11.9 (.6)^f^	9.4 (.7)^g^
20% lactic acid^b^	12.2 (.7)^h^	10.1 (.6)^i^
30% lactic acid^c^	6.1 (.6)^j^	4.1 (.5)^k^
50% lactic acid^d^	5.1 (.5)^l^	3.1 (.6)^m^
37% phosphoric acid^e^	8.2 (.6)^n^	6.2 (.6)^o^

*SD: standard deviation.

Different superscript numbers represent  significant differences between the shear bond strengths of each storage time (*P* < .001). Different lowercase letters represent significant differences between the etchant ∗ storage times within each group (*P* < .001).

## References

[1] Amra I, Samsodien G, Shaikh A, Lalloo R (2007). Xeno III self-etching adhesive in orthodontic bonding: the next generation. *American Journal of Orthodontics and Dentofacial Orthopedics*.

[2] Keizer S, Ten CJM, Arends J (1976). Direct bonding of orthodontic brackets. *American Journal of Orthodontics*.

[3] Lopez JI (1980). Retentive shear strengths of various bonding attachment bases. *American Journal of Orthodontics*.

[4] Miura F, Nakagawa K, Masuhara E (1971). A new direct bonding system for plastic brackets. *American Journal of Orthodontics and Dentofacial Orthopedics*.

[5] Reynolds IR (1985). A review of direct orthodontic bonding. *British Journal of Orthodontics*.

[6] Wang WN, Yeh CL, Fang BD, Sun KT, Arvystas MG (1994). Effect of H_3_PO_4_ concentration on bond strength. *Angle Orthodontist*.

[7] Sheen DH, Wang WN, Tarng TH (1993). Bond strength of younger and older permanent teeth with various etching times. *Angle Orthodontist*.

[8] Justus R, Cubero T, Ondarza R, Morales F (2010). A new technique with sodium hypochlorite to increase bracket shear bond strength of fluoride-releasing resin-modified glass ionomer cements: comparing shear bond strength of two adhesive systems with enamel surface deproteinization before etching. *Seminars in Orthodontics*.

[9] Dorminey JC, Dunn WJ, Taloumis LJ (2003). Shear bond strength of orthodontic brackets bonded with a modified 1-step etchant-and-primer technique. *American Journal of Orthodontics and Dentofacial Orthopedics*.

[10] Buyukyilmaz T, Usumez S, Karaman AI (2003). Effect of self-etching primers on bond strength—are they reliable?. *Angle Orthodontist*.

[11] Paskowsky TN (2003). Shear bond strength of a self-etching primer in the bonding of orthodontic brackets. *American Journal of Orthodontics and Dentofacial Orthopedics*.

[12] Buonocore MG (1955). A simple method of increasing the adhesion of acrylic filling materials to enamel surfaces. *Journal of Dental Research*.

[13] Newman GV (1965). Epoxy adhesives for orthodontic attachments: progress report. *American Journal of Orthodontics*.

[14] Galil KA, Wright GZ (1979). Acid etching patterns on buccal surfaces of permanent teeth. *Pediatric Dentistry*.

[15] Carstensen W (1992). The effects of different phosphoric acid concentrations on surface enamel. *Angle Orthodontist*.

[16] Hannig M, Reinhardt KJ, Bott B (1999). Self-etching primer vs phosphoric acid: an alternative concept for composite-to-enamel bonding. *Operative Dentistry*.

[17] Fritz UB, Diedrich P, Finger WJ (2001). Self-etching primers—an alternative to the conventional acid etch technique?. *Journal of Orofacial Orthopedics*.

[18] Miller RA (2001). Laboratory and clinical evaluation of a self-etching primer. *Journal of Clinical Orthodontics*.

[19] Shimada Y, Senawongse P, Harnirattisai C, Burrow MF, Nakaoki Y, Tagami J (2002). Bond strength of two adhesive systems to primary and permanent enamel. *Operative Dentistry*.

[20] Ermis RB, de Munck J, Cardoso MV (2008). Bond strength of self-etch adhesives to dentin prepared with three different diamond burs. *Dental Materials*.

[21] van Dijken JW (2004). Durability of three simplified adhesive systems in Class V non-carious cervical dentin lesions. *American Journal of Dentistry*.

[22] Al-Omari WM, Mitchell CA, Cunningham JL (2001). Surface roughness and wettability of enamel and dentine surfaces prepared with different dental burs. *Journal of Oral Rehabilitation*.

[23] Kidd EAM (1976). Microleakage : a review. *Journal of Dentistry*.

[24] Sano H, Yoshikawa T, Pereira PN (1999). Long-term durability of dentin bonds made with a self-etching primer, *in vivo*. *Journal of Dental Research*.

[25] Armstrong SR, Keller JC, Boyer DB (2001). The influence of water storage and C-factor on the dentin-resin composite microtensile bond strength and debond pathway utilizing a filled and unfilled adhesive resin. *Dental Materials*.

[26] Gwinnett AJ, Yu S (1994). Effect of long-term water storage on dentin bonding. *American Journal of Dentistry*.

[27] Santerre JP, Shajii L, Leung BW (2001). Relation of dental composite formulations to their degradation and the release of hydrolyzed polymeric-resin-derived products. *Critical Reviews in Oral Biology and Medicine*.

[28] Britton JC, McInnes P, Weinberg R, Ledoux WR, Retief DH (1990). Shear bond strength of ceramic orthodontic brackets to enamel. *American Journal of Orthodontics and Dentofacial Orthopedics*.

[29] Canay S, Kocadereli I, Akca E (2000). The effect of enamel air abrasion on the retention of bonded metallic orthodontic brackets. *American Journal of Orthodontics and Dentofacial Orthopedics*.

[30] Urabe H, Rossouw PE, Titley KC, Yamin C (1999). Combinations of etchants, composite resins, and bracket systems: an important choice in orthodontic bonding procedures. *Angle Orthodontist*.

[31] Barkmeier WW, Erickson RL (1994). Shear bond strength of composite to enamel and dentin using Scotchbond Multi-Purpose. *American Journal of Dentistry*.

[32] Gardner A, Hobson R (2001). Variations in acid-etch patterns with different acids and etch times. *American Journal of Orthodontics and Dentofacial Orthopedics*.

[33] Yamada R, Hayakawa T, Kasai K (2002). Effect of using self-etching primer for bonding orthodontic brackets. *Angle Orthodontist*.

[34] Çehreli ZC, Altay N (2000). Effects of a non-rinse conditioner and 17% ethylenediaminetetra acetic acid on the etch pattern of intact human permanent enamel. *Angle Orthodontist*.

[35] Mount GJ, Clark JW (1984). Glass ionomer cements: clinical consideration. *Clinical Dentistry*.

[36] Geoff D (1992). Lactic acid is your friend. *Bicycling*.

[37] Bell GH, Davidson JN, Enslie-Smith D (1976). *Textbook of Physiology and Biochemistry*.

[38] Ayad MF, Rosenstiel SF, Farag AM (1996). A pilot study of lactic acid as an enamel and dentin conditioner for dentin-bonding agent development. *Journal of Prosthetic Dentistry*.

[39] Shinchi MJ, Soma K, Nakabayashi N (2000). The effect of phosphoric acid concentration on resin tag length and bond strength of a photo-cured resin to acid-etched enamel. *Dental Materials*.

[40] Silverstone LM, Saxton CA, Dogon IL, Fejerskov O (1975). Variation in the pattern of acid etching of human dental enamel examined by scanning electron microscopy. *Caries Research*.

